# Get up early: Revealing behavioral responses of sandeel to ocean warming using commercial catch data

**DOI:** 10.1002/ece3.8310

**Published:** 2021-11-26

**Authors:** Ole Henriksen, Anna Rindorf, Henrik Mosegaard, Mark R. Payne, Mikael van Deurs

**Affiliations:** ^1^ National Institute for Aquatic Resources Technical University of Denmark Kgs Lyngby Denmark

**Keywords:** climate change, dormancy, emergence, fish, North Sea, phenology, temperature

## Abstract

1. Warming of the oceans and shifts in the timing of annual key events are likely to cause behavioral changes in species showing a high degree of site fidelity. While this is well studied in terrestrial systems, there are fewer examples from the marine environment. Sandeel (*Ammodytes marinus*) is a small eel‐shaped teleost fish with strong behavioral attachment to sandy habitats in which they are buried from late summer through winter. When spring arrives, the sandeel emerge to feed during the day for several of months before returning to the sand for overwintering refuge.
2. Using fisheries data from the North Sea, we investigated whether catch rates reflect the timing of emergence and if seasonal patterns are related to temperature and primary production.
3. Catch per unit effort (CPUE) was used to describe sandeel emergence. We developed indicators of the relative timing of the emergence from the winter sand refuge and the subsequent growth period. Different modeling approaches were used to investigate the relationship with bottom temperature and primary production.
4. Variation in emergence behavior was correlated with variation in sea bottom temperature. Warmer years were characterized by earlier emergence. Significant warming over the last three decades was evident in all sandeel habitats in the North Sea throughout most of their adult life history, though no net shift in the phenology of emergence was detected. Minimum temperature during spring was a better predictor of emergence behavior than, for example, degree days.
5. This study emphasizes how temperature‐induced changes in behavior may have implications for predators and fisheries of sandeel. The method can be applied to other species for which the timing of exploitation (i.e., fisheries) and species life history are well matched.

## Graphical abstract

The fishery for sandeels in the North Sea comprise several species from the Ammodytidae family. Although, the majority of catches comprises the lesser sandeel (*Ammodytes marinus*, smaller individual), other species such as the great sandeel (*Hyperoplus lanceolatus*, larger individual) also occur in the fishery. Thus, the findings from this study might also be valid and relevant for the other species (also worth mentioning, *A. tobianus*, *H. immaculatus* and *Gymnammodytes semisquamatus*) that co‐occur with the lesser sandeel in some areas of the North Sea.

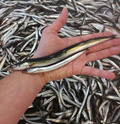

## 1 INTRODUCTION

Temperature is an essential driver of a variety of ecosystem dynamics (Parmesan, 2006; Parmesan & Yohe, 2003; Root et al., 2003) and the general expectation is that warming of temperate and arctic regions will dampen seasonality or shift the timing of transitions between seasons (Burrows et al., 2011; Edwards & Richardson, 2004; Menzel et al., 2006). While the behavioral responses of animals to such changes are well documented in terrestrial systems (Davis et al., 2010; Ge et al., 2015; Ovaskainen et al., 2013), they are less studied in aquatic systems, where focus has been placed mainly on migratory species (Hollowed et al., 2013; Peer & Miller, 2014; Sims et al., 2004). Aquatic organisms can either “adapt, move, or die” in response to warming. Mobile fish can migrate either vertically to deeper waters or horizontally to other areas, whereas species that are constrained by, for example, habitat requirements may need to adapt behaviorally or physiologically to temperature changes (Baker, 2021; Kleisner et al., 2017; Roessig et al., 2004).

One mechanism by which organisms can adapt their life cycle to varying availability of food and changes in temperature is diapause, a state of dormancy where development and/or metabolism slows (Hand et al., 2016). Diapause typically precedes the onset of sustained unfavorable environmental conditions (e.g., winter or dry season), maintaining the organism in a dormant state until exposure to specific cues or stimuli (Speers‐Roesch et al., 2018). Fish belonging to the Ammodytidae family (i.e., sandeels or sand lances) exhibit a burying behavior for diapause, presumably as a mechanism to cope with cold periods, food shortage, and possibly high predation. The exact dormancy mechanism (e.g., hibernation vs. winter dormancy) seem to be poorly studied and undecided for these teleost fish (Soyano & Mushirobira, 2018). Currently the scientific census point toward that colder water species of the Ammodytidae family (e.g., *Ammodytes marinus*, *Ammodytes hexapterus*, *Ammodytes personatus*, *Ammodytes americanus*, and *Ammodytes dubius*) enter a low‐temperature dormancy, also termed overwintering, buried in cold periods (Baker et al., 2019; Staudinger et al., 2020; van Deurs et al., 2010; Winslade, 1974c), whereas the warmer water species (*Ammodytes japonicus* and *Ammodytes heian*) enter a high‐temperature dormancy, also termed aestivation, buried in the warm periods (Kim et al., 2017; Kuzuhara et al., 2019; Tomiyama & Yanagibashi, 2004). Lesser sandeel *A. marinus* in the North Sea (hereafter referred to as sandeel) shows strong site fidelity (Gauld, 1990; Jensen et al., 2011; Wright et al., 2019) and bury in the sediment from late summer through winter, relying mainly on stored energy reserves (MacDonald et al., 2018; van Deurs et al., 2010, 2011). When spring arrives, the sandeel emerge from the sand to feed and grow rapidly for a couple of months (van Deurs et al., 2013). They feed in shoals during the day and retreat to the sand during the night (Engelhard et al., 2008; Freeman et al., 2004). The success of this strategy relies on the balance between food intake during the feeding period, energy expenditure during the dormant period, and a timely spring emergence (Baker et al., 2019; van Deurs et al., 2010). As sandeel are most vulnerable to predators when entering and leaving the sediment (Hobson, 1986; Johnsen et al., 2017; Temming et al., 2004, 2007), premature emergence or unnecessary protraction of the feeding period may result in an increased predation mortality. If emergence of sandeel in the North Sea, as well as Ammodytidae species in other ecosystems, is stimulated by environmental triggers, such as temperature and food production (van Deurs et al., 2010, 2011; Winslade, 1974a, 1974c), ocean warming may lead to shifts in the timing of emergence, and such shifts in phenology are likely to impact the many predators (e.g., fish, seabirds and mammals) for which these species constitute a substantial fraction of their diet (de Boer, 2010; Engelhard et al., 2014; Furness, 2002; Gilles et al., 2016; Greenstreet et al., 1998; Harris & Wanless, 1991; Sharples et al., 2009).

**FIGURE 1:**
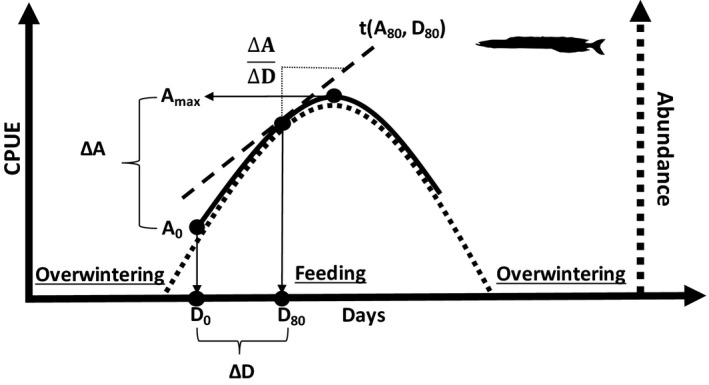
Conceptual figure illustrating the theoretical framework for how commercial catch rates (CPUE as a proxy for abundance) inform about the biomass of sandeel. The biomass of sandeel follows in theory a dome‐shaped curve, where sandeel become available to the predators and the fishery during a feeding (and growth) period (dashed line and dashed *y*‐axis). Sandeel biomass gradually increase as fish emerge from overwintering and start growing, and then gradually decline as feeding ceases and the sandeel once again spend more time submerged in the sand. Assuming CPUE is proportional to the biomass, it will follow a similar curve (solid line and solid *y*‐axis). Note that an even distributed dome‐shape is for illustration purpose. Thus, the curve can vary in shape with the slope and function of the increasing and decreasing trends in biomass during emergence and around the onset of overwintering, respectively. *D*_0_ is the first day of fishing (a fixed day), *A*_0_ is the sandeel biomass on the first day of fishing, and *A*_max_ is the maximum biomass. The difference between *A*_max_ and *A*_0_ (Δ*A*) was used in this study as a measure of the relative timing of emergence, where a low value indicates early emergence. Timing of emergence was also approximated by the time difference between *D*_0_ and the day where 80% of *A*_max_ is reached (*D*_80_)

Here we explored the possibility of using commercial catch data to identify phenological responses (i.e., timing of spring emergence) in sandeel. The North Sea sandeel is targeted by a large industrial fishery in the emergence period (Dickey‐Collas et al., 2014; Furness, 2002; Nielsen & Mathiesen, 2006; Nielsen, 1989). We expected that the development of biomass catch rates in this fishery would follow a distinct dome‐shaped pattern over time, reflecting the seasonal time window of feeding and growing (Engelhard et al., 2008; Reeves, 1994) (Figure 1). We designed a set of quantitative indicators describing timing of spring emergence and tested the prediction that temperature or food availability explains variation in emergence behavior. Based on initial investigations (Henriksen, 2020), and previous studies (Winslade, 1974c), sandeel activity associated with emergence are expected to be triggered by proximate stimuli when residing in the sand, and therefore, sea bottom temperature were chosen as an adequate proxy. We first screened for correlations between emergence behavior and either monthly sea bottom temperature or monthly phytoplankton concentrations (as a measure of food production). Hence, two hypotheses were tested: (1) the increase in food availability in spring “wakes up” the sandeel (*food trigger hypothesis*) and (2) temperature acts as trigger stimuli that “wakes up” the sandeel (*temperature trigger hypothesis*). This first screening identified temperature as the most promising predictor of the timing of emergence, which motivated further testing of alternative temperature predictors to advance our understanding of the underlying mechanisms. This lead to the inclusion of yet a third hypothesis: (3) temperature acts indirectly via a bioenergetics pathway, where depletion of energy stores is what “wakes up” the sandeel. Descriptions of working hypotheses and predictors are summarized in Table 1.

**TABLE 1:** Detailed summary of the different hypotheses and associated predictors used in models

Hypothesis	Predictor details	Predictor abbreviation	Model
Food trigger hypothesis	Monthly averages of phytoplankton	PP	Linear model
Temperature trigger hypothesis	Monthly averages of observed sea bottom temperature	SBT	Linear model
Temperature trigger hypothesis (instant trigger)	Day for minimum observed sea bottom temperature	Day_min_	Linear mixed model
Temperature trigger hypothesis	Minimum observed sea bottom temperature	*T*_min_	Linear mixed model
Temperature trigger hypothesis	Rate of spring warming	Slope_s_	Linear mixed model
Temperature trigger hypothesis	Heating degree days after day for minimum observed SBT in spring	HDD	Linear mixed model
Bioenergetics trigger hypothesis	Degree days integrated over the overwintering period (September–April)	DD_ow_	Linear mixed model
Bioenergetics trigger hypothesis	Degree days integrated in winter around spawning (December–February)	DD_w_	Linear mixed model

## 2 MATERIALS AND METHODS

### 2.1 Catch data

**FIGURE 2:**
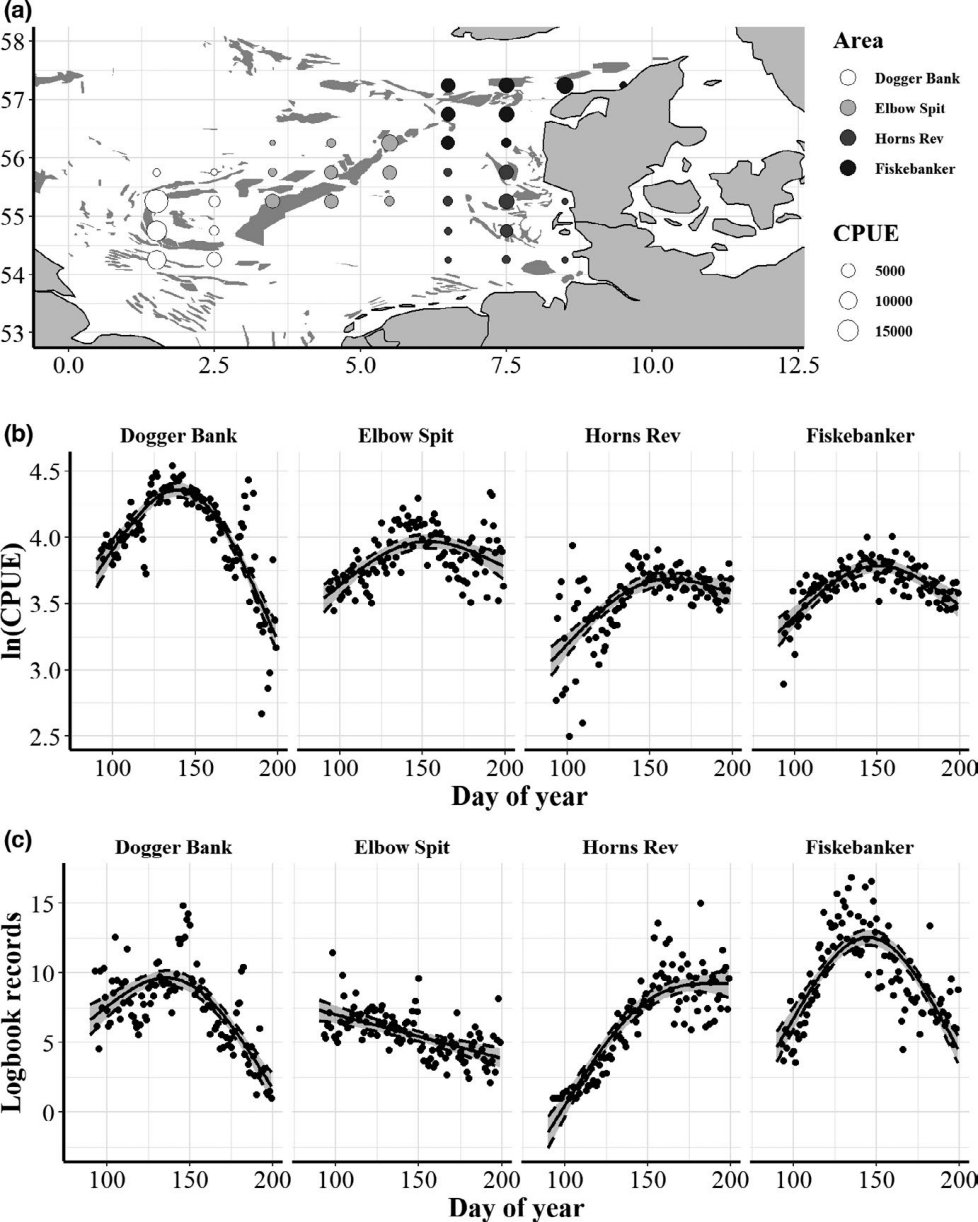
Overview over catch data in the four areas selected for the study (a), average dynamics in commercial catch rates (natural logarithm of daily CPUE) over the fishing season (b), and average logbook records over the fishing season (c). Sandeel habitats reproduced from Jensen et al. (2011) are also shown (dark gray sand banks) and CPUE are summed by ICES statistical rectangles (circles in the center of each rectangle). Each area is shown separately and the predicted smooth trend lines with confidence intervals for CPUE and logbook records averaged across all years were produced using general additive models (*k* = 3). The four study areas were Dogger Bank (white circles), Elbow Spit (light gray circles), Horns Rev (dark gray circles), and Fiskebanker (black circles)

Biomass catch rates were derived from logbook records of the Danish industrial fishery. The fishery predominately use herded volume otter trawls modified for small pelagic fish (Eigaard et al., 2011, 2016) and sandeel are targeted when they are active in the water column during day. Catches (in ton) and effort (numbers of fishing days) were extracted from individual logbook records from 1992 to 2018 together with information about the statistical rectangles (1 longitude × 0.5 latitude) fished. Sufficient spatial and temporal data coverage for the analysis was ensured by only considering rectangles within four major fishing areas: Dogger Bank, Elbow Spit, Fiskebanker, and Horns Rev (Figure 2a). Today, the fishery is conducted between April 1 and August 1, but in the 1990s, a fishery targeting 0 groups took place after August 1. Zero‐group fish arrive at the fishing grounds in late June and remain in the water column after the older age groups have begun overwintering. Data after July 19 (200th day of the year) were therefore excluded to avoid confounding the analyses with data from the autumn 0‐group fishery (Figure 2b). As the data are not derived from organized scientific surveys, the coverage in space and time varies. The starting date was therefore required to have a minimum of three logbook records on average to avoid that single logbook records which obtained a high weight (e.g., outlier catches from one vessel) in the analyses (Figure 2c). As fishing tends to start later in the coastal areas of Fiskebanker and Horns Rev, only data after 93rd (April 2) and 121st (May 2) days of the year, respectively, were included for these areas.

### 2.2 Standardization of catch per unit effort

The average size of vessels has increased substantially throughout the time series and smaller vessels tend to start the season followed by larger vessels. As larger vessels have higher biomass catch rates even when fishing in the same area at the same time, it was necessary to correct catch rates for differences in vessel size. This was done by standardizing all effort data (number of fishing days) to days equivalent to a 200‐gross registered tonnage vessel using a vessel size correction factor *b* estimated using the following general formulation of catch biomass per unit effort (CPUE):

$${\text{CPUE}}_{V,i}=\frac{C_{i}}{E_{V,i}}=q_{0}V^{b}B,$$

where $C_{i}$ is the recorded catch in logbook record *i* and $E_{V,i}$ is the effort of vessel size *V* (gross tonnage) in logbook record *i*. *q*_0_ denotes the catchability of a standard vessel (and is thus independent of changes in size composition in the fleet) and *B* is biomass. *b* was estimated through mixed models having year *y*, ICES statistical rectangle *s* and week *w* as mixed effects in four separate periods (1989–1998, 1999–2005, 2006–2016, 2017–2019). The approach assumes that catches within the same rectangle, week, and year are taken at the same biomass *B*. In addition, individual observations of catches by a single vessel were assumed similar to those taken by the same vessel in other areas and times (i.e., ID, vessel effect). The specification of each mixed model was as follows:

$$\ln\left({\text{CPUE}}_{w,s,y,V,\text{ID}}\right)=\varphi_{w,s,y}+\lambda_{\text{ID}}+b*\ln\left(V\right),$$

where $\varphi_{w,r,y}$ and $\lambda_{\mathrm{ID}}$ are separate normal distributed parameters, each with a mean of 0. Residuals were examined for signs of nonlinearity in the relationship between CPUE and *V*, but none were found. There was a tendency for over‐occurrence of large negative residuals in all periods. Standardized effort was estimated as:

$$E_{200,i}={\left(\frac{V}{200}\right)}^{b}E_{V,i}$$

Hence, as *b* is greater than zero, a fishing day of a vessel larger than 200 tonnage will result in a standardized fishing effort of more than one day, whereas a day of fishing on a vessel smaller than 200 tonnage results a standardized fishing effort of less than one day. Catches were unchanged.

### 2.3 Models of seasonal patterns in sandeel biomass

**FIGURE A1:**
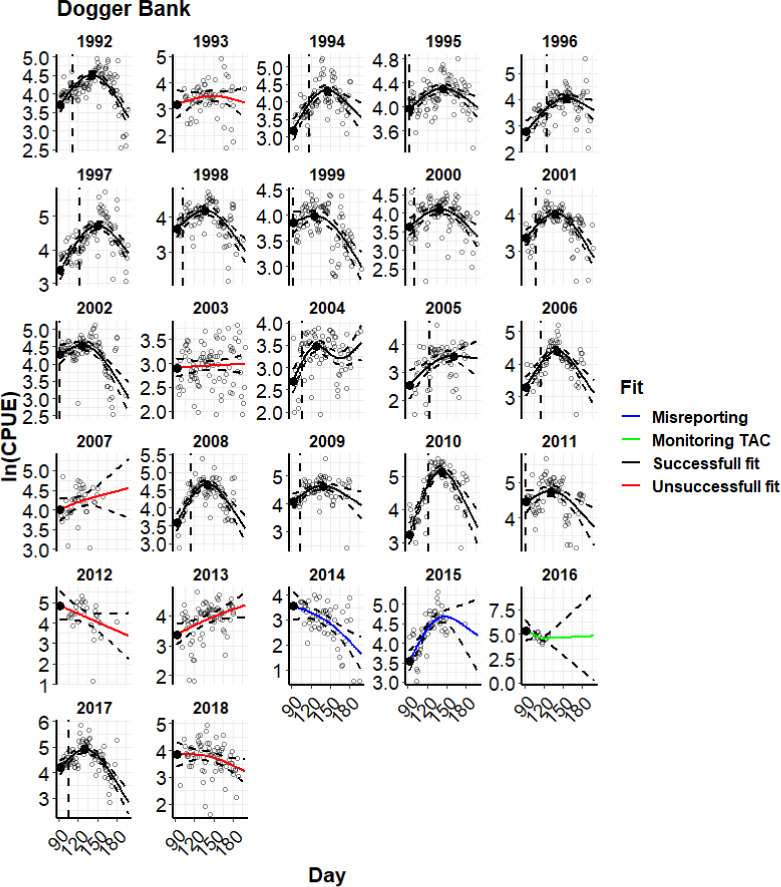
GAM fits for abundances (i.e., ln[CPUE]) as a function of days for Dogger Bank. Based on a number of criteria, successful fits (black lines) are kept in the analysis. Unsuccessful fits (red lines) as well as years with misreporting (blue lines) and monitoring TACs (green lines) are shown, but were excluded from the analysis. Estimated *A*_0_ and *A*_max_ (black dots, see also Figure 1) and *D*_80_ (dashed vertical line) are indicated for all successful fits

**FIGURE A2:**
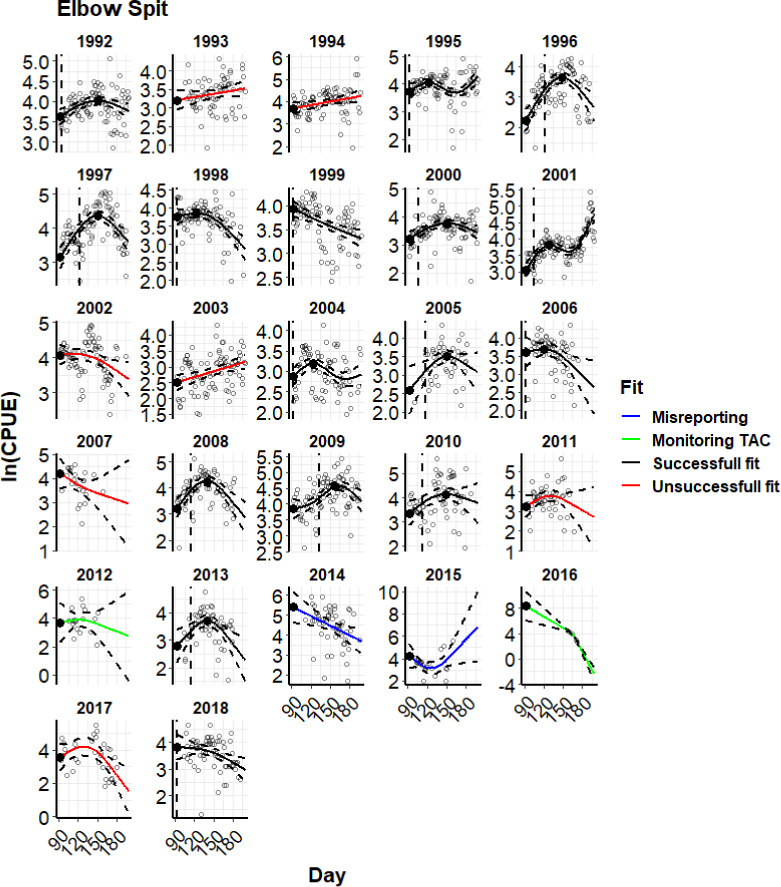
GAM fits for abundances (i.e., ln[CPUE]) as a function of days for Elbow Split. Based on a number of criteria, successful fits (black lines) are kept in the analysis. Unsuccessful fits (red lines) as well as years with misreporting (blue lines) and monitoring TACs (green lines) are shown, but were excluded from the analysis. Estimated *A*_0_ and *A*_max_ (black dots, see also Figure 1) and *D*_80_ (dashed vertical line) are indicated for all successful fits

**FIGURE A3:**
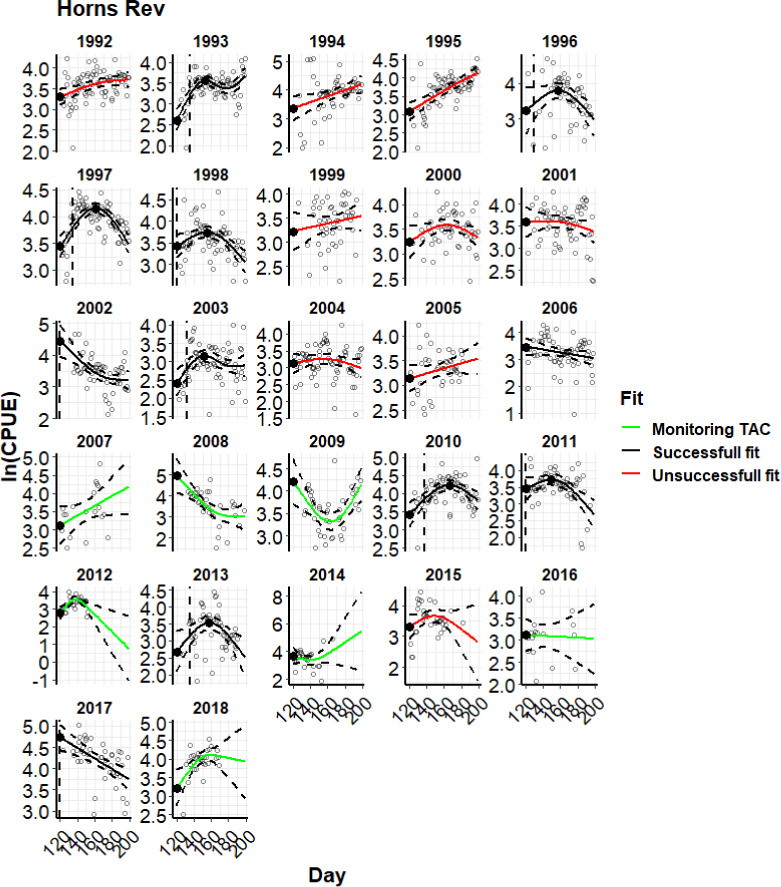
GAM fits for abundances (i.e., ln[CPUE]) as a function of days for Horns Rev. Based on a number of criteria, successful fits (black lines) are kept in the analysis. Unsuccessful fits (red lines) as well as years with monitoring TACs (green lines) are shown, but were excluded from the analysis. Estimated *A*_0_ and *A*_max_ (black dots, see also Figure 1) and *D*_80_ (dashed vertical line) are indicated for all successful fits

**FIGURE A4:**
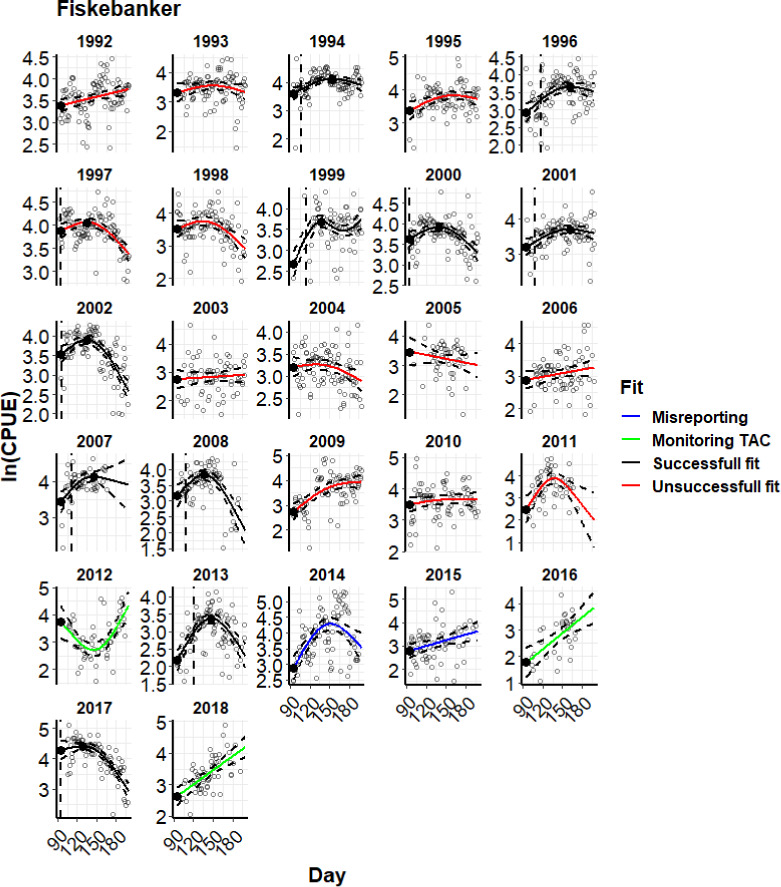
GAM fits for abundances (i.e., ln[CPUE]) as a function of days for Fiskebanker. Based on a number of criteria, successful fits (black lines) are kept in the analysis. Unsuccessful fits (red lines) as well as years with misreporting (blue lines) and monitoring TACs (green lines) are shown, but were excluded from the analysis. Estimated *A*_0_ and *A*_max_ (black dots, see also Figure 1) and *D*_80_ (dashed vertical line) are indicated for all successful fits

We used CPUE as an indicator for daytime biomass. To calculate the predicted CPUE on any given day and test the expectation that CPUE conforms to a dome‐shaped pattern (Figure 1), general additive models (GAM) were fitted to the log‐transformed daily CPUE values (restricting the number of knots to 3). Separate GAMs were fitted for each year and area. If the fitted curve reached the maximum at the first or the last data point in the model (i.e., no dome‐shaped pattern emerged), the number of knots was increased from 3 to 4 (to force a dome‐shaped pattern). Recognizing that the data were associated with substantial noise and that the choice of when and where to fish is influenced by the behavior of the fishermen and fisheries management, some data quality criteria were applied: GAMs which explained <10% of the variance in the CPUE data or where the maximum of the curve was predicted to be at the last data point were excluded from further analysis. GAM fits from years where catches were <10,000 ton and years with <15 data points after day 150 were also excluded (i.e., years with highly limited quotas or fishery closures), because too few data points late in the season prevented reliable dome‐shaped fits (see Figure 2b). Finally, we also excluded years with substantial misreporting (ICES, 2018). About half of the year–area combination passed the data quality criteria. All GAM fits (including the discarded year–area combinations) are shown in the Figures A1, A2, A3, A4.

### 2.4 Indicators of emergence

The fitted GAM curves were used to derive emergence indicators. A variety of indicators of emergence were considered in the study. Initial analyses showed that the dates of the earliest logbook records in a given year were not a robust indicator of the timing of emergence. Overall fishing effort have decreased since 2003 and the season opening date has been regulated, resulting in a delay of the first logbook record that is likely to be unrelated to emergence. Furthermore, in several years, CPUE was relatively high when the fishing commenced, indicating that the onset of emergence occurred before the first logbook record. As the actual emergence was not observed, we instead used the development in CPUE over time to derive three indicators.

The first indicator (Δ*A*) was the relative change in CPUE (commercial catch rates as a proxy for abundance) from the first day of fishing to the predicted maximum, estimated as ln(*A*_max_/*A*_0_), where *A*_0_ and *A*_max_ are the predicted CPUE from the GAM fit on the first day of fishing and the predicted maximum CPUE, respectively (Figure 1). If *A*_max_ and *A*_0_ are influenced mainly by the overall stock biomass and timing of emergence, it can be assumed that Δ*A* is small in years where onset of emergence happened early and vice versa. However, Δ*A* may also be influenced by the rate of emergence and growth rate. The third indicator therefore described the slope of the increase in CPUE (see description below).

**FIGURE 3:**
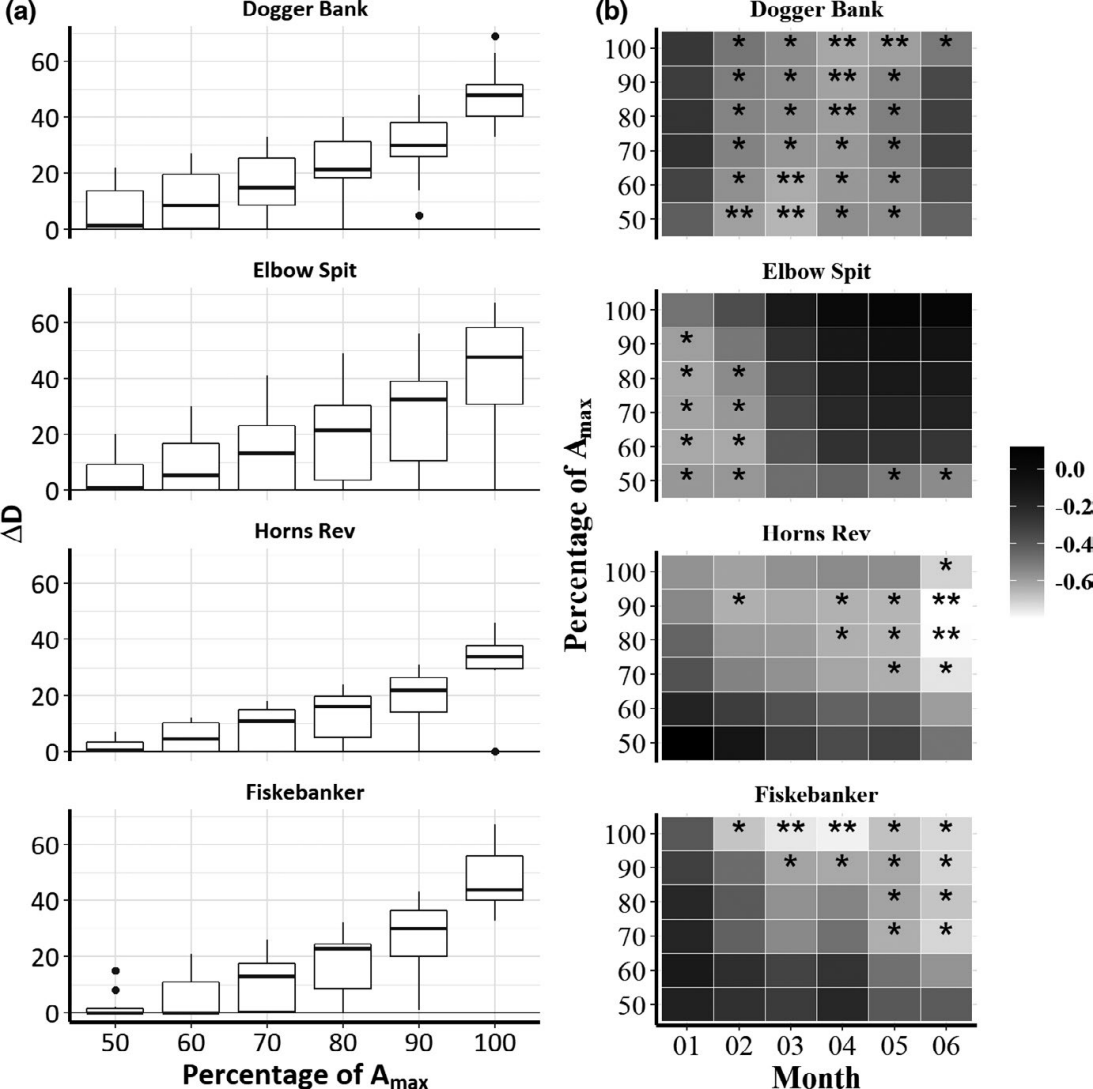
Sensitivity analysis of estimated days of timing of emergence (Δ*D*). Different threshold values estimated as percentage (50%, 60%, 70%, 80%, 90%) of *A*_max_ (100%) were investigated. Box plots (a) and Pearson's correlation statistics with SBT (b) are shown. Color gradient represents Pearson's correlation coefficient and significance levels are indicated by stars (*p < .05, **p < .01, ***p < .001)

**FIGURE A5:**
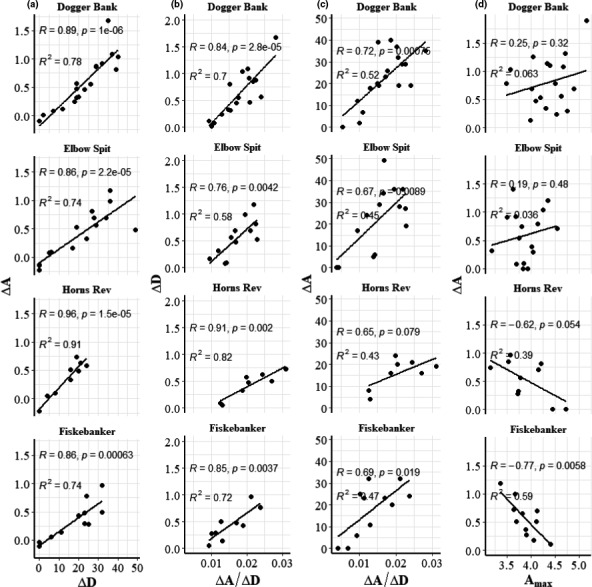
Relationship between timing of emergence indicators Δ*A* versus Δ*D* (a), Δ*D* versus Δ*A*/Δ*D* (b), Δ*A* versus Δ*A*/Δ*D* (c), and Δ*A* versus *A*_max_ (d). Lines are fitted using simple linear regressions. Explained variation (*R* ^2^) are provided, as well as Pearson's correlation coefficient (*R*) and *p* value (*p*)

The second indicator (Δ*D*) represented the time difference between the day at which CPUE was 80% of *A*_max_ (*D*_80_) and the first day of fishing (*D*_0_) (Figure 1). Preliminary sensitivity analyses revealed that above 80% of *A*_max_ yielded the most robust results when compared to lower percentages of *A*_max_ (Figure 3a). The most conservative percentage was chosen assuming that there was a risk of overshooting when approaching *A*_max_ (100%). Assuming the rate of emergence is constant, a small Δ*D* indicates early emergence. We expected Δ*A* and Δ*D* to be correlated (Figure A5).

The third indicator (Δ*A*/Δ*D*) reflected the slope of the tangent (*t*) to the CPUE curve in the point defined by [*D*_80_, *A*_80_] (Figure 1). This indicator reflects the rate of increase in emerged biomass just prior to *A*_max_, which is an indicator of growth rate and rate of emergence, but not timing of emergence. Hence, we expect Δ*A* and Δ*D* to be correlated to Δ*A*/Δ*D* only if the two former indicators are reacting to changes in emergence rate and individual growth (Figure A5). Furthermore, if interannual variation in, for example, temperature influences individual growth and not timing of emergence, we expect to find correlations between Δ*A*/Δ*D* and temperature and not between Δ*A* or Δ*D* and temperature and vice versa.

### 2.5 Temperature and phytoplankton data

We obtained measures of sea bottom temperature (SBT, °C, 1992–2018) and concentration of phytoplankton (PP, 0–10 m depth, mgC m^−3^, 1998–2018) from the UK MetOffice issued under the European Commission (Copernicus website: http://marine.copernicus.eu/). Initial investigations found that measures of sea surface temperatures were highly correlated with SBT (Henriksen, 2020), and thus the most proximate stimuli for temperature was decided to be sufficient for the current analysis. The data are the results of the Atlantic‐European North‐West Shelf‐Ocean Physics Reanalysis (NORTHWESTSHELF_REANALYSIS_PHY_004_009) and the Biogeochemistry Reanalysis (NORTHWESTSHELF_REANALYSIS_BIO_004_011), respectively. Data were downloaded as daily values in a grid of longitudinal–latitudinal 7 km^2^ cells, and from that we calculated averages (averaging across grid cells) for all four study areas (Figure 2a and Figure A7). Sandeel feed on zooplankton, but due to the lack of a reliable zooplankton index we used PP as a proxy for zooplankton productivity.

Monthly averages of SBT and PP were calculated for all years in each area to achieve an overall impression of whether emergence was driven by SBT or PP (i.e., contesting the temperature hypothesis and the food hypothesis). Thereafter, a series of alternative temperature predictors were calculated to further advance our understanding of the underlying mechanisms. Minimum SBT (*T*_min_) was calculated as the average temperature of the 10 days surrounding the day of minimum SBT (Day_min_) in spring (March–May). Heating degree days (HDD) were computed as the sum of daily SBTs from Day_min_ to the last day in May. The slope, or rate, of the increase in SBT during spring (Slope_s_) was calculated as the average of the tangents to the SBT slope on (i.e., first derivative) March 1, April 1, and May 1, respectively.

Finally, degree days for the entire overwintering dormant period (DD_ow_, September–April) and for the winter spawning period (DD_w_, December–February) were calculated. A description of the working hypotheses associated with the different temperature predictors is provided in Table 1.

### 2.6 Statistical models

To investigate the support in the data for the food hypothesis versus temperature trigger hypothesis, we used simple linear regressions:

I∼a+bM,

where *I* is the emergence indicator (Δ*A*, Δ*D*, or Δ*A*/Δ*D*), *a* is the intercept, and *b* is the estimated slope of the predictor *M* (monthly SBT or PP). Separate regressions were conducted for each of the first 5 months of the year (January–May) and each of the areas.

To further investigate the different alternative temperature‐related hypotheses (Table 1), we combined data from all four areas in the same linear mixed‐effect model:

I∼a+bT+ε,

where *T* represents the temperature predictor (Day_min_, *T*_min_, Slope_s_, DD_ow_, or DD_w_) and the random effect of area on the intercept is given by $\varepsilon ∼N\left(0,\sigma^{2}\right)$. Separate models were fitted for each of the five temperature predictors. Furthermore, for each of the four areas, normal linear regression models were also fitted for each area in order to inform about the area‐specific differences.

Temperature (average SBT) trends over the study period were quantified with mixed linear models (SBT∼a+bYear+ε) in all areas during each month in autumn (September–November), winter (December–February), spring (March–May), and summer (June–August).

## 3 RESULTS

**FIGURE 4:**
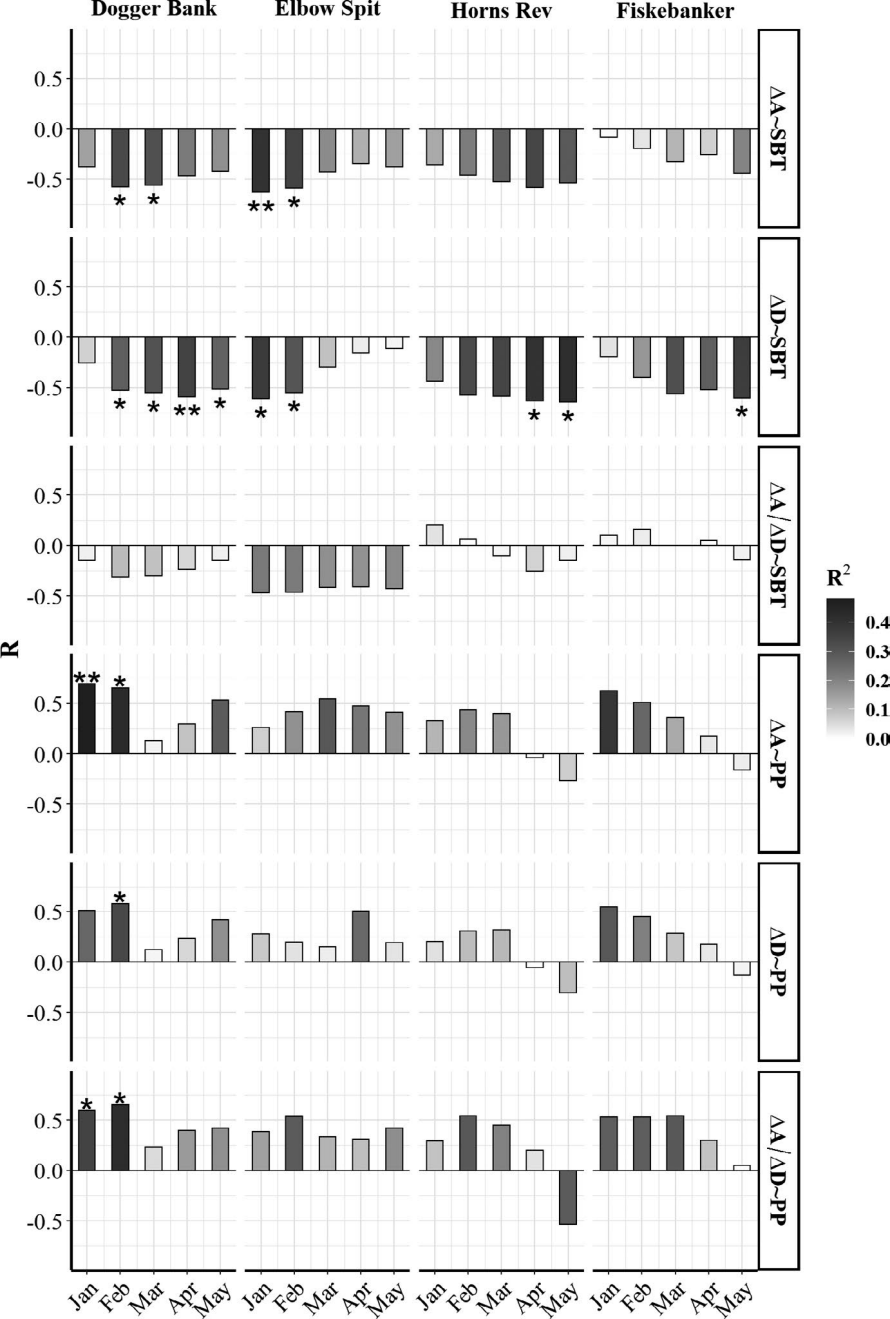
Model statistics for simple linear regression models (model formulation in right panel) using a monthly averages of sea bottom temperature (SBT) and phytoplankton (PP) to explain indicators of timing of emergence Δ*A*, Δ*D*, and growth (Δ*A*/Δ*D*). Both the correlation coefficient (*R*, bars) and coefficient of determination (*R* ^2^, color) are shown for all linear models for each area (Dogger Bank, Elbow Spit, Horns Rev, and Fiskebanker, upper panel) and each month (January–May). All slope estimates for overall average fits (*) are associated with *p* values (*p < .05, **p < .01)

Of the year–area combination, 62% passed the data quality criteria (see Figures A1, A2, A3, A4) and all remaining GAM fits displayed some sort of dome shape in accordance with our expectations (Figure 1). However, the shape and the explanatory power of the GAM fits differed substantially between areas and years. The overall highest degree of explained variation and most distinct dome shapes were found for Dogger Bank (18 of 24 GAM fits passed the quality criteria), where the GAM fits explained 30.5% of the variance on average. The fitted GAMs in other areas had lower explanatory power on average (Fiskebanker, 28.3%; Horns Rev, 21.8%; Elbow Spit, 20.0%) and less years passed the quality criteria due to issues related to, for example, catch limits and/or monitoring quotas (Elbow Spit, 16 of 23; Horns Rev, 11 of 20; Fiskebanker, 10 of 22). Most areas reached peak CPUE in April except Horns Rev that peaked 1 month later in May (*D*_80_; Elbow Spit at day 111.37 ± 15.88 SD, Fiskebanker at day 112.82 ± 11.71 SD, Dogger Bank at day 114.56 ± 12.05 SD, and Horns Rev at day 132.80 ± 9.01 SD). This difference between areas was mainly due to a delayed fishing season also observed as a late increase in vessels on Horns Rev (Figure 2c). Based on the confidence intervals, the uncertainty around fitted curves and metrics (*A*_0_ and *A*_max_) used for estimating timing of emergence in abundance (Δ*A*) were generally low (see Figures A1, A2, A3, A4). The sensitivity analysis that explored different thresholds for estimating timing of emergence in days (Δ*D*) showed that at low percentages of *A*_max_ (50–70%), the risk of underestimating days since emergence (i.e., a given threshold becomes equal to *D*_0_) were high (Figure 3a). Choosing *D*_80_ for subsequent analysis represented the most conservative threshold for further analysis. Choosing higher percentages as a threshold would increase the level of significance, but not the explanatory power for relationships with SBT (Figure 3b), as well as in the relationship with other temperature predictors used (analysis not shown, but see predictors in Table 1 and Figure 4).

In accordance with the temperature hypothesis, decreasing relationships between monthly SBT and emergence indicators Δ*A* and Δ*D* were observed in all months in all areas, indicating later emergence in cold years (Figure 4). Emergence indicators on Dogger Bank showed significant relations with SBT in 2 or more months (Δ*A*, ${\bar{R}}^{2}$ = 0.32; Δ*D*, ${\bar{R}}^{2}$ = 0.30) (Figure 4). Elbow Spit showed significant relationships in 2 months for both indicators (Δ*A*, ${\bar{R}}^{2}$ = 0.37; Δ*D*, ${\bar{R}}^{2}$ = 0.34). Horns Rev and Fiskebanker only showed significant relationships for Δ*D* (Δ*D*, ${\bar{R}}^{2}$ = 0.48; Δ*D*, ${\bar{R}}^{2}$ = 0.36), but the level of significance was dependent on the threshold choice (Figure 3b). PP showed significant relationships (Δ*A*; ${\bar{R}}^{2}$ = 0.45; Δ*D*; ${\bar{R}}^{2}$ = 0.34) only on Dogger Bank (Figure 4) and the relationship was positive, which counterintuitively indicates that a large phytoplankton production would delay emergence.

**FIGURE 5:**
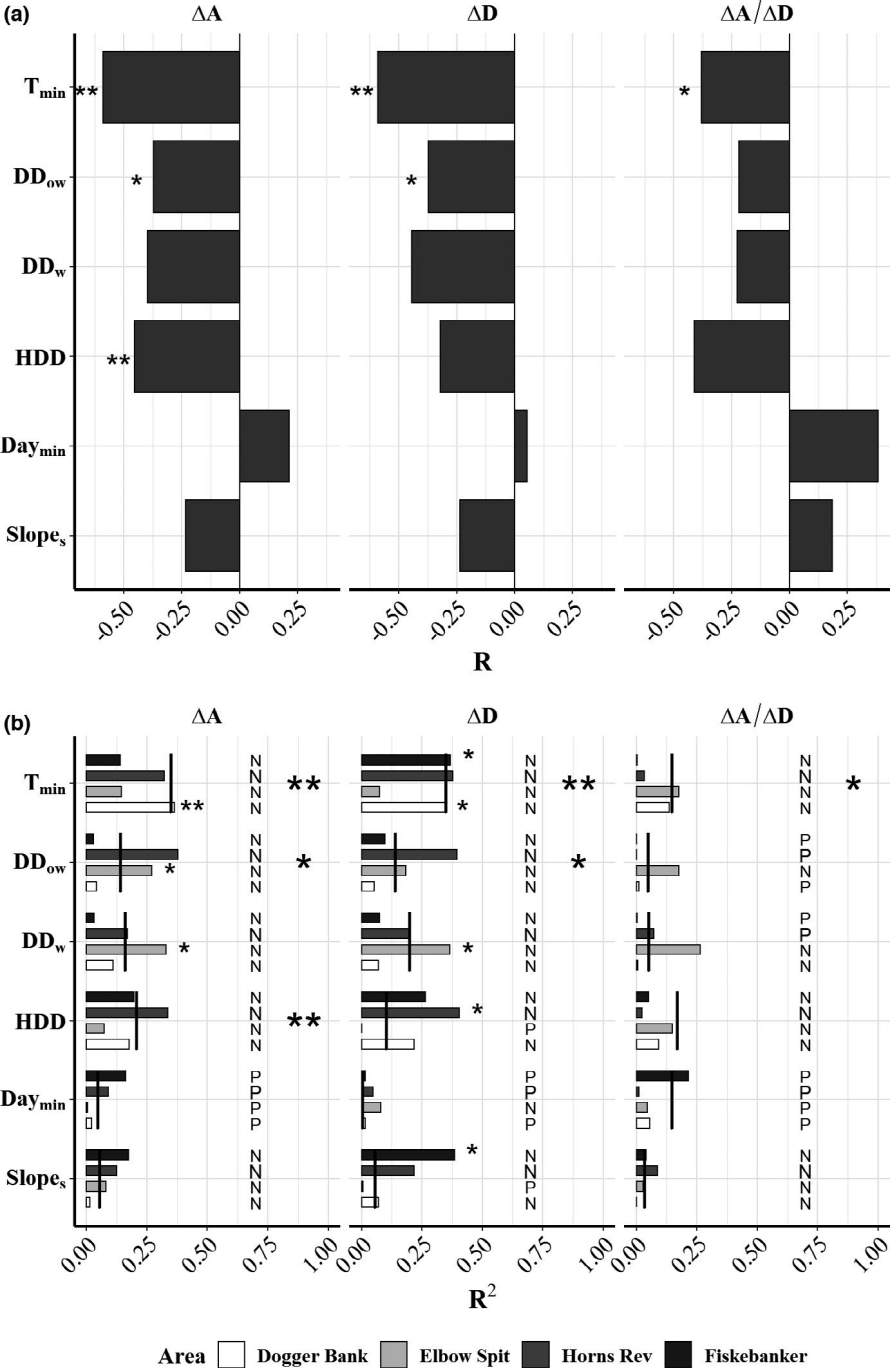
Model statistics for models using a series of temperature predictors to explain indicators of timing of emergence Δ*A* (left), Δ*D* (middle), and growth (Δ*A*/Δ*D*, right). The temperature predictors were calculated using sea bottom temperature (SBT). The correlation coefficient (*R*) for mixed‐effects regression models using all data with area as a random effect for the overall average fit (a) and coefficient of determination (*R* ^2^) for area‐specific linear regressions (b) are shown. For each area, normal linear models are fitted to data (colored bars: Dogger Bank [white], Elbow Spit [light gray], Horns Rev [dark gray], and Fiskebanker [black]). *R* ^2^ for mixed models (black lines) presented in (a) are shown for comparison. Positive (P) and negative (N) slope effects are annotated for all linear models. All slope estimates for overall average fits and for area‐specific linear models are associated with *p* values (*p < .05, **p < .01, ***p < .001)

The mixed‐effect models used to compare the performance of the alternative temperature predictors and associated hypothesis (Table 1) revealed that the overall most consistent predictions of emergence was achieved with *T*_min_ (Figure 5a). This predictor showed significant negative relationships with both Δ*A* and Δ*D* (Δ*A*, *R* ^2^ = 0.35; Δ*D*, *R* ^2^ = 0.35, Figure 5b). The second best predictor was DD_ow_, but the relationships were weaker and less significant. HDD were significantly related to Δ*A* (*R* ^2^ = 0.20), but not to Δ*D* (Figure 5a). Day_min_, DD_w_, and Slope_s_ were not significantly related to the emergence indicators. Overall, the linear mixed‐effect models showed poor relationships between temperature predictors and Δ*A*/Δ*D* (Figure 5a), indicating that temperature mainly affected emergence timing. Area‐specific estimates from simple linear regressions of the relationships between temperature predictors and emergence indicators revealed that the relationships with *T*_min_ and HDD were robust in at least three areas, whereas DD_ow_ were driven by mainly two areas (Figure 5b).

**FIGURE 6:**
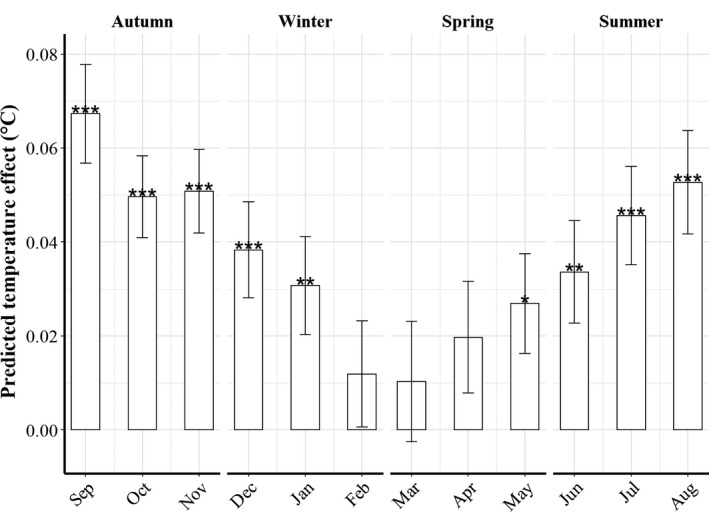
Model predicted temperature effects (monthly averages of SBT ± SE) over the entire study period. Temperature trends were quantified using mixed‐effect linear models for all months (including area as a random effect). Positive predicted temperature effects indicate increasing trends. The *p* values for the slope effects are indicated by * (*p < .05, **p < .01, ***p < .001)

**FIGURE A6:**
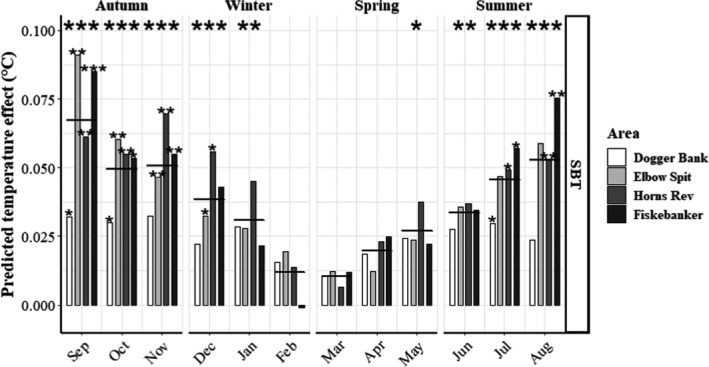
Model predicted temperature effects (°C) for linear regressions using year as a predictor. The yearly slope effect of temperature is shown for all months during autumn, winter, spring, and summer for the sea bottom temperature (SBT). For each area, normal linear models are fitted to data (colored bars: Dogger Bank [white], Elbow Spit [light gray], Horns Rev [dark gray], and Fiskebanker [black]). Linear mixed models (black lines) presented in Figure 5 are shown for comparison using all data with area as a random effect for the overall average fit. All slope estimates for area‐specific linear models (small *) and overall average linear mixed models (large *) are associated with *p* values (*p < .05, **p < .01, ***p < .001)

Temperature increased significantly over time (between 1992 and 2018) in most months, but not in February, March, and April (Figure 6). No significant temporal trend was detected for Δ*A* and Δ*D* over the study period (linear mixed regression, *p* > .05). Warming was generally more pronounced in coastal areas of Horns Rev and Fiskebanker, whereas the effect was weaker in Elbow Spit and Dogger Bank (Figure A6).

## 4 DISCUSSION

Biomass catch rates conformed to a dome‐shaped pattern with increasing catch rates until a peak, followed by declining catch rates. This enabled us to develop emergence indicators and test if timing of emergence was influenced by other factors than photoperiod, such as temperature or food. The results suggested that emergence after dormancy takes place earlier in years with relatively high sea bottom temperatures in winter and spring. In contrast, phytoplankton concentrations showed less consistent relationships with emergence indicators and the relationships were mainly positive, which indicated that large phytoplankton production would delay emergence. The present results support the temperature trigger hypothesis, although based solely on these analyses, the role of food for sandeel emergence remains unresolved.

Among the different temperature predictors, the minimum temperature (*T*_min_) yielded the most robust results. However, degree days calculated for the dormancy period (DD_ow_) also showed significant effects on both emergence indicators. The temperature increased significantly over time between 1992 and 2018 in most months, except for February, March, and April, and since *T*_min_ is most often found to be in March, this may explain why the emergence indicators did not display a temporal trend over time toward gradually smaller values (i.e., earlier emergence). Thus, global warming seems not to be driving the emergence patterns for sandeel.

Data were more noisy at low catch rates and for the dome‐shaped patterns of emergence that were less well defined. Even though bimodal patterns could be observed in the data, these patterns are likely not reflecting the emergence of sandeel, but rather area‐specific behavior and dynamics governed by the fishery. For example, increases in CPUE due to high catches of young individuals at the start of the time series, as well as shifts in the distribution of fisheries that can allocate and concentrate fishing effort in specific areas during the season, might be causing such bimodal patterns. Furthermore, fisheries advice and management can drive years of low CPUE, which were often excluded from the analysis based on the predefined quality criteria. This underlines the importance of having access to high‐quality catch data from fisheries with relatively stable fishing behavior and management. The curation of fishery datasets to account for bias and standardizing effort have been highlighted in several studies (Bishop, 2006). Accounting for vessel effects and time series differences (i.e., model standardization of CPUE) as well as fishery dynamics (i.e., exclusion of data based on fishery information) were important for this study. We suggest that our method can be applied to other commercial fish species for which high‐quality data are available and the timing of fisheries and species life history are well matched. For example, fisheries for *Ammodytes* spp. in the Mediterranean and Japan could be interesting cases to investigate for comparison (Dickey‐Collas et al., 2014; Matsuda et al., 2010; Maynou et al., 2021). On that note, while methods and results presented here only were designed to detect whether emergence behavior were sensitive to temperature, the analyses do not account for nonlinear responses to temperature. Future work could apply alternative approaches modeling nonlinearity and account for spatial variation in a singular model framework (Ciannelli et al., 2012), which might capture variation that inform on other aspects of the emergence dynamics.

Despite the lack of robust relationships between phytoplankton and emergence indicators, the potential role of food availability should not be discarded based on this study alone. Phytoplankton, averaged over larger areas, showed low predictive power of emergence and thus do not provide any insight into sandeel emergence. Although our analysis was designed to capture the lag phase between spring phytoplankton bloom and zooplankton peak abundance, the linear relationships were not able to capture such dynamics. This might reflect several limitations to the methods, including, for example, the linear model design, the resolution and overlap in data sources, noise inherent to fishery‐dependent data, and the difficult challenges of drawing connections across multiple system scales. Overall, we suspect that the measure of phytoplankton used in our study performed poorly as a proxy for important zooplankton prey for sandeel. Large copepods are important for efficient foraging (van Deurs et al., 2014, 2015), where specifically two species of *Calanus* play important roles for sandeel in the North Sea (Régnier et al., 2017; van Deurs et al., 2009). These notions are in line with growth estimates from modeling bioenergetics (MacDonald et al., 2018; van Deurs et al., 2015). Growth of prey copepods is temperature controlled (Edwards & Richardson, 2004; Møller et al., 2012). When water temperatures during winter are relatively high, spring temperatures optimal for copepod growth may be reached sooner than in years with cold winters. In that case, temperature would merely be a secondary trigger acting via the copepods. Food as a driver of sandeel activity has also been shown for *Ammodytes tobianus* in laboratory experiments (van Deurs, Behrens, et al., 2011). However, time series of the relevant prey field and feeding conditions are difficult to obtain, and though the continuous plankton recorder (Capuzzo et al., 2018) would have been one alternative way to include more direct measures of zooplankton, deciding on which zooplankton species to include and how to weigh their relative importance with a relevant temporal and spatial resolution were considered outside the scope of the study.

**FIGURE A7:**
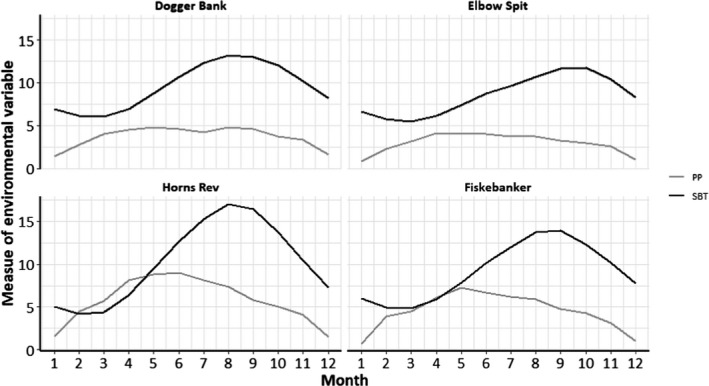
Average monthly concentration of phytoplankton (PP, <10 m depth, mgC m^−3^, gray line) and temperature at the sea bottom (SBT, °C, black line) for all areas

The level of swimming activity of North Sea sandeel reach a minimum around 5°C and activity increases from 5 and 10°C (Winslade, 1974c). In our study, the average temperatures across all areas in April and May were within this temperature range (~6.3–8.4°C), except for Horns Rev, where temperatures in most years exceeded 10°C in May. Furthermore, bottom temperatures, when abundance of sandeel in the water column peaks on Dogger Bank in May, are in the range of ~8.3–9.0°C (this study and van der Kooij et al., 2008). Thus, the temperature increase from March to May could play an important role in triggering emergence and feeding activity. Nonetheless, if it is the rate of increase in temperature during spring that determines when the sandeel emerge, then the temperature predictors termed Slope_s_ and HDD should have been better correlated to the emergence indicators, which was not the case. Instead, the global mixed‐effect model (i.e., model with area as a random effect) showed that *T*_min_ explained more of the variation in the emergence indicators than any other temperature predictor tested. There may, however, be area‐specific differences. The main effects (detected using separate linear regression models for different months and areas) were found during winter and early spring in offshore areas (i.e., Dogger Bank and Elbow Spit), whereas late spring seemed to contribute more in the coastal areas (i.e., Fiskebanker and Horns Rev). These differences in the spatial response may be attributed to much larger temperature span in the coastal areas, where average bottom temperatures rise from below 5°C, suggested to be the minimum threshold for swimming activity in sandeel (Winslade, 1974c), in March to above 10°C in late May. In comparison, the bottom temperature on Dogger Bank is never below 5°C on average and the increase are limited to a couple of degrees between March and May (Figure A7). Whether these differences can be attributed to adaptive behavior in sandeel and/or fisheries behavior remain unresolved, though the latter are expected to drive much of the variation. For example, the fishery at Horns Rev starts approximately 20 days later than in other areas and thus the delayed fishing season are likely to explain the later peak in abundance observed. Furthermore, choosing a threshold higher than 80% of *A*_max_ would change the perception of the relationship with Δ*D* observed at Fiskebanker to be prevalent already from late winter. Therefore, the area‐specific relationships obtained by simple linear models should be evaluated with caution, but further research that pursue the spatial relationships are encouraged. Nevertheless, the notion that temperature is determining swimming activity and transitions between dormancy and active periods has not only been suggested for sandeel in the North Sea (Winslade, 1974c), but has been proposed for a related species, *A. japonicus* (Tomiyama & Yanagibashi, 2004), which points toward temperature as an essential driver of both onset and release from dormancy in teleost fish from the Ammodytidae family. Thus, the findings from this study are highly relevant for other species of sandeels in the North Sea (Figure 8). Furthermore temperature has been highlighted as a general mechanism determining dormancy in other teleost fish (Silva et al., 2019; Speers‐Roesch et al., 2018). However, also variation in photoperiod could potentially explain some of the interannual variation in the emergence indicators, especially during winter and early spring when photoperiod remain low and increased cloud cover are prevalent (Campbell et al., 2008; Hut & Beersma, 2011; Winslade, 1974b).

Besides the direct influence on swimming activity, temperature may also act via bioenergetic pathways. DD_ow_ provides a measure of the temperature regime experienced by the sandeel during the dormancy period of overwintering and may therefore serve as a proxy for energy expenditure during dormancy. DD_ow_ was the second best predictor of emergence, indicating that bioenergetics and depletion of energy reserves may be part of the underlying mechanism. For example, warm winters result in premature depletion of energy reserves and emergence. However, since energy depletion is not only a function of temperature, but also body size, a more detailed investigation of the bioenergetics hypothesis would require the inclusion of time series of size and ideally also bioenergetics modeling. Although, initial investigations leading to the current study explored the latter (Henriksen, 2020), it was considered outside the framework of this paper. In support of the bioenergetics hypothesis, previous studies have demonstrated relationships between recruitment survival and size of age 0 fish (Foy & Paul, 1999; Henriksen et al., 2021), and both theoretical and experimental work have demonstrated accelerated reserve depletion during warm winters (van Deurs, Hartvig, et al., 2011; Wright et al., 2017).

Individual weight of sandeel in the North Sea increase during spring when they start feeding (*c*. 60% weight gain, Rindorf et al., 2016) and varies substantially between years (range of ±40% based on data in ICES, 2018), corresponding to around 0.5 on a natural logarithmic scale. Hence, changes over the season in catch rates of sandeel are a measure of the combined effects of emergence from an inactive overwintering phase and subsequent somatic growth. The observed increase in CPUE (i.e., caught biomass per unit effort) over the season greatly exceeds what can be explained by growth alone, and hence we are confident that the dome shape of the GAM fits also reflects emergence dynamics. Furthermore, Δ*A*/Δ*D* was less strongly correlated to the temperature predictors than was Δ*A* and Δ*D*, suggesting that temperature influenced mainly the timing of emergence. However, Δ*A*/Δ*D* was correlated with Δ*A* and Δ*D*, respectively, suggesting that Δ*A* and Δ*D* were not independent of the rate of emergence and weight gain (Figure A5).

This study contributes to the more general field of research endeavoring to understand the interplay between photoperiod and other triggers, such as temperature, in fine‐tuning the timing of seasonal events (Bradshaw & Holzapfel, 2010). One of the major challenges in this field of research is to distinguish between photoperiod and temperature, because we need time series of information about when spring activity commences. Many studies can therefore only report an approximate temperature and date, without being able to disentangle the effect of interannual variation in temperature regimes (Christoffersen et al., 2019; Westerberg & Sjöberg, 2015). This study therefore also proposes a novel way of acquiring such time series that potentially could be applied to other commercially harvested fish species with strong seasonal activity cycles. Furthermore, the merits of this work show potential for integrating factors into forecasting (Christensen et al., 2013; Henriksen et al., 2018), where temperature possibly can be used to predict emergence of sandeel, which might be useful as an early warning for the fisheries industry.

This study found significant relationships that suggested how warming in of bottom waters in spring near sandy habitats, which are used as overwintering refuge for sandeel, may trigger the emergence from winter dormancy. Hence, in years with high spring temperature, the sandeel emerge to feed earlier. While temperatures significantly increased in most months over time during the study period, months in late winter and early spring temperatures showed no significant increase. In support, the lack of a temporal pattern in emergence might point that global warming is decoupled from the emergence behavior of sandeels. If early emergence is synonym with shorter periods of dormancy and prolonged activity periods, this could in turn result in increased predation mortality and a miss‐balanced trade‐off between energy gain and survival probability (van Deurs et al., 2010). Shifts in the timing of emergence relative to timing of the fishery may also affect fishing mortality and pose a problem to the harvest output, if the targeted biomass comprised smaller energy‐depleted individuals with reduced economic value (Dickey‐Collas et al., 2014; von Biela et al., 2019). Moreover, sandeel exert tremendous grazing pressure on the zooplankton (van Deurs et al., 2013), and in the North Sea, several species of seabirds are relying on sandeel as a food resource (Burthe et al., 2012; Frederiksen et al., 2011; Wanless et al., 2018). Similar strong links between predators and availability of *Ammodytes* spp. have been highlighted in other ecosystems (Baker et al., 2019; Goyert, 2015; Ito et al., 2012; Staudinger et al., 2020; Suca et al., 2021; Tomita et al., 2009), thus changes in the timing of sandeel emergence could potentially have ecosystem‐wide consequences affecting plankton communities, predators, and the fisheries opportunities. However, despite the scientific advancement made in this study, the exact mechanisms underpinning the link between temperature and emergence remain unknown and further studies are needed on this topic.

## CONFLICT OF INTEREST

The authors declare no conflict of interest.

## AUTHOR CONTRIBUTIONS

**Ole Henriksen:** Conceptualization (lead); Formal analysis (lead); Investigation (lead); Methodology (lead); Visualization (lead); Writing‐original draft (lead); Writing‐review & editing (lead). **Anna Rindorf:** Conceptualization (supporting); Data curation (equal); Formal analysis (supporting); Funding acquisition (equal); Investigation (supporting); Methodology (supporting); Project administration (equal); Resources (equal); Supervision (equal); Visualization (equal); Writing‐review & editing (equal). **Henrik Mosegaard:** Conceptualization (supporting); Funding acquisition (equal); Investigation (supporting); Methodology (supporting); Project administration (equal); Resources (equal); Supervision (supporting); Validation (supporting); Visualization (supporting); Writing‐review & editing (supporting). **Mark R. Payne:** Conceptualization (supporting); Formal analysis (supporting); Funding acquisition (supporting); Investigation (supporting); Methodology (supporting); Resources (supporting); Supervision (supporting); Visualization (supporting); Writing‐review & editing (supporting). **Mikael van Deurs:** Conceptualization (equal); Formal analysis (equal); Funding acquisition (equal); Investigation (equal); Methodology (equal); Project administration (lead); Supervision (lead); Validation (equal); Visualization (equal); Writing‐review & editing (equal).

## Data Availability

Regrettably, our institute has a General Data Protection Regulation which is valid for certain data sources, such as fisheries vessel data. They are classified as sensitive personal data, where vessels potentially can be backtracked to individual vessels. Thus, the data used for the article will be shared on reasonable request to the institute or corresponding author.
